# Self-focused or other-focused: The influence of acknowledgment type on subsequent donation desires

**DOI:** 10.3389/fpsyg.2022.959369

**Published:** 2022-09-23

**Authors:** Feng Wenting, Shen Xianyun, Yin Zuowei

**Affiliations:** ^1^Gemmological Institute, China University of Geosciences, Wuhan, China; ^2^Research Center for Psychological and Health Sciences, China University of Geosciences, Wuhan, China

**Keywords:** self- *vs*. other-focused acknowledgment, subsequent donation behavior, morality preference, charitable organizations, acknowledgment type

## Abstract

This study employs morality preference theory to explore how acknowledgment type (self- *vs*. other-focused) influences donors' subsequent donation desires. The current research consists of 3 studies. Study 1 finds that an other-focused acknowledgment letter elicits higher subsequent donation desires than a self-focused letter. Study 2 testifies to the mediating role of morality preference between the relationship of acknowledgment type and subsequent donation desires. Study 3 manipulates the moral value on “what is the morally right thing of donation” and developed a new scale to measure morality preference. Study 4 excludes the influence of language structure and tests the main effect in a real donation behavior context.

## Introduction

The sustainability of charitable organizations crucially depends on maintaining donors (Sargeant and Woodliffe, 2007). Most new donors disappear soon after their first-time donations, which leads to a huge waste of charitable resources (Sargeant, 2001; Burk, 2003). Therefore, charities exert themselves to establish a long-term relationship with donors (Gundlach et al., 1995; Bennett and Barkensjo, 2005; Shabbir et al., 2007; Bennett, 2009).

Charities are recommended to build a lasting relationship with donors by acknowledging them (Bennett, 2006; Merchant et al., 2010). Normally, two approaches are employed by charities to express their gratitude toward these donors. One emphasizes the altruistic benefits of donation behaviors, which is called an other-focused acknowledgment. The other type focuses on admiring the personal qualities of donors, which is a self-focused acknowledgment. Does the acknowledgment type influence subsequent donation desires? The current research focuses on this question.

Prior research reveals that acknowledgments can encourage prosocial behaviors in donors (Kotler and Lee, 2005; Grace and Griffin, 2006). However, this positive effect is affected by several moderating variables, such as the occasions (Kotler and Lee, 2005), the source of acknowledgment (Wenting et al., 2021), external or private rewards (McCullough et al., 2001), and events or individual characteristics (Winterich et al., 2013b). Does the acknowledgment type (self-focused or other-focused) influence subsequent donation desires? Previous studies have been unable to provide a reasonable answer to this question.

To remediate this theoretical gap, the current study investigates the impact of acknowledgment type (self-focused or other-focused) on subsequent donation desires according to morality preference theory (Tappin and Capraro, 2018). The current research consists of 3 studies. Study 1 indicates that the acknowledgment type plays a significant role in subsequent donation desires. Study 2 tests the mediating role of morality preference in the relationship between acknowledgment type and subsequent donation desires. Study 3 manipulates the moral value on “what is the morally right thing of donation” to verify the mediating effect in a further step. Study 4 excludes the influence of language structure and tests the main effect in a real donation behavior context.

Prior study shows that the information type (positive, or negative) provided by charity influences donation performances (Erlandsson et al., 2018). The intuitive information (images of identified victims) and the rational information (information about charity efficiency and effectiveness) also influence subsequent donation performances. To exclude these effects, the current research adds several tests to the studies. Previous research also finds the mediating role of emotional reactions, perceived impact, and perceived responsibility in helping situations (Erlandsson et al., 2015). To rule out these possible mediating effects, the current research conducts a multi-mediation analysis in study 2.

The current research first distinguishes other-focused acknowledgments and self-focused acknowledgments, enriches relevant studies in the research field of acknowledgment, constructs an integrative model for the proposed hypothesis, and provides effective suggestions for charities. Secondly, this study also explores the influences of other- and self-focused frames on donation desires in the acknowledgment context. Other- *vs*. self-focused appeals may trigger different motivations that lead to varying donation behavior. The current research focuses on subsequent donation desires, which provides a different context for other- *vs*. self-focused frames. Finally, based on morality preference theory, the current study offers an explicit explanation of donors' complex attitudes toward acknowledgments.

## Literature review and hypothesis development

### “Self- *vs*. other-benefit” charitable appeals

Charitable appeals have been categorized as a type of public service ad (Bagozzi and Moore, 1994). Research in this area has examined the behavioral responses to appeals for helping oneself and others (Bagozzi and Moore, 1994). White and Peloza (2009) define two different kinds of charitable appeals: “other-benefit” appeals that highlight that the main beneficiary of support is some other individual or organization; and “self-benefit” appeals that highlight that the main beneficiary of support is the donor (Brunel and Nelson, 2000; White and Peloza, 2009).

Both other-benefit and self-benefit appeals are commonly studied by marketing researchers and used by charitable organizations (Bendapudi et al., 1996; Brunel and Nelson, 2000; Ferguson et al., 2008; Fisher et al., 2008). For example, White and Peloza (2009) indicate that other-benefit (self-benefit) appeals generate more favorable donation support than self-benefit (other-benefit) appeals in situations that heighten (*vs*. minimize) public self-image concerns. Feiler et al. (2012) discovered that emphasizing both egoistic and altruistic reasons in charitable appeals reduces donors' donation desires. Brunel and Nelson (2000) find that females prefer ads that focus on helping others to those that focus on helping oneself, while males prefer self-directed ads to ads directed toward helping others.

However, prior studies have not explored the influence of the “other” and “self” frames in the context of acknowledgments. Although charitable appeals and acknowledgment letters are both channels for charities to reach people, there are significant differences between them. Charitable appeals act as an advertisement to encourage people to provide donations, particularly in the case of attracting new donors. In contrast, acknowledgment letters are used as a reinforcer to improve the desire of existing donors to make subsequent donations. Therefore, the current research explores how acknowledgment type (“other” and “self”) influences donors' subsequent donation desires according to morality preference theory.

### Type of acknowledgment

Acknowledgments are employed by charities or organizations to give positive attention to helpers (Kwarteng et al., 1988). There exists a significant distinction among acknowledgments. Some acknowledgments focus on other people and emphasize the altruistic benefits of donation behaviors. Some acknowledgments focus on the donor and emphasize the positive qualities of the donors. Charities often express their appreciation of donors in two different ways. For example:

“Your donation means a lot to those in need and gives them hope in life! Thank you very much for your donation.”

It can also be expressed like this:

“We truly appreciate your kindness and generosity. This is a praiseworthy virtue and a good deed! Thank you very much for your donation.”

The first type of acknowledgment mainly highlights the impact of donation behavior on others, for example, the benefits to others, which this study defines as an other-focused acknowledgment. The second type emphasizes the influence of donation behavior on self, such as the good qualities of the donor, which is called a self-focused acknowledgment. Building on morality preference theory, the current study investigates the influence of acknowledgment type (self- *vs*. other-focused) on subsequent donation desires.

### Morality preference

Morality refers to the social norms that regulate the relationships among people, society, and nature. People engage in various activities by moral standards, which can bring positive influences on society and individuals (Haidt and Kesebir, 2010). When a person accepts a certain moral norm, the external moral norm becomes the individual's moral value. Moral value is the internal standard held by individuals when they judge whether various social phenomena conform to morality. It is the sum of various moral norms recognized by individuals. Moral values can indicate what is the ethical way of behavior and regulate a person's behavior (Haidt and Kesebir, 2010). Morality preference refers to a kind of innate behavioral tendency, that is, people prefer to do the behavior in line with moral values (Bicchieri, 2005; DellaVigna et al., 2012; Huck et al., 2012). If something is consistent with an individual's moral value and triggers an individual's feelings of doing the morally right thing, it increases the individual's tendency to do it (Capraro et al., 2022). Therefore, the current research argues that morality preference includes three important elements: consistency with moral values (cognition), the feeling of doing the morally right thing (perception), and moral tendencies in behavior (intention).

Morality preference is an important motivation for prosocial behavior. A great deal of research has sought to understand what motivates individuals to be pro-social. Behavioral economists typically assume that people have social preferences that minimize inequality and/or maximize efficiency (social welfare) (DellaVigna et al., 2012). However, pro-social individuals tend to choose the option that is perceived as morally correct in a given situation, rather than the outcome of a comprehensive assessment of all options, whether the outcome is fair or efficient (Capraro and Rand, 2018). Lots of findings support the idea of moral preferences, whereby individuals broadly derive “doing the right thing” rather than being particularly concerned with efficiency and fairness. The premise of morality preference is that individuals derive utility from performing actions they perceive to be morally right (Bicchieri, 2005; DellaVigna et al., 2012; Huck et al., 2012; Krupka and Weber, 2013; Tappin and Capraro, 2018). This perspective accords with evidence from social psychology that individuals derive utility from seeing themselves in a positive moral light (Aquino and Reed, 2002; Dunning, 2007). It is moral factors, rather than fairness or efficiency, that influence individuals' behavioral preferences (Capraro et al., 2022). Altruistic behavior in the dictator game and cooperative behavior in the prisoner's dilemma is partly driven by a desire to do the morally right thing (Capraro and Rand, 2018; Tappin and Capraro, 2018). To sum up, morality preference is an important driver of people's altruistic behavior. Altruistic behavior is generally in line with moral values, which can trigger the perception of doing the morally right thing.

Morality preference also can be influenced by external environment and stimuli. First, strengthening moral values can enhance moral preference in individuals. For example, morality preference has been used to increase prosocial behavior by simply using interventions that make the morality of an action salient (Capraro and Vanzo, 2019; Capraro et al., 2019; Capraro and Perc, 2021). Capraro et al. (2019) found that moral nudges (e.g., making moral norms salient) can promote prosocial behavior. Capraro et al. (2019) also found that asking people to report what they think is the morally right thing to do, which increases crowdsourced charitable donations by 44%. Moral reminders increase prosocial behavior in both the dictator game and the prisoner's dilemma (Brañas-Garza, 2007; Dal Bó and Dal Bó, 2014; Capraro et al., 2019). Second, changing the perceptions of individuals on what is morally right thing can lead to different behavioral tendencies. Krupka and Weber (2013) find that framing effects in dictator games vary due to changes in perceptions of individuals on what is morally right. Behavior in the trade-off game is highly sensitive to the labels used to describe the available actions. This framing effect could be explained by a change in what people think to be the morally right thing to do (Eriksson et al., 2017; Capraro and Rand, 2018). Capraro and Rand (2018) find that the majority of people chose the option framed as morally appropriate.

In conclusion, morality preference is one of the most important internal motivations for individuals' prosocial behaviors. Individuals' feeling about doing the morally right thing will significantly affect their donation behaviors. Therefore, the current research employs the morality preference theory to explore the influence of acknowledgment type on donation behavior.

### The influence of acknowledgment type on donation behavior

In the current research, based on the morality preference theory, acknowledgment type (self-vs. other-focused) will influence subsequent donation desires. Specifically, according to the morality preference theory, individuals derive utility from performing actions they perceive to be morally right. An other-focused acknowledgment emphasizes the altruistic benefits of donation behaviors that act as moral nudges. It provides positive feedback from the beneficiary and is directed at the altruistic perceptions of the donor, which conforms to moral values and increases the donor's feeling of doing the morally right thing (Capraro and Rand, 2018; Capraro et al., 2019). An other-focused acknowledgment focuses on donation behavior as meaningful and beneficial for the recipient (Baumeister and Leary, 1995; Elliott et al., 2005). The donor realizes that assistance that helps others is altruistic and moral behavior, which elicits the donor's moral preference and increases subsequent prosocial behavior. Morality preference plays an important internal motivation in prosocial behavior that improves subsequent donation desires (Capraro and Rand, 2018; Tappin and Capraro, 2018). Therefore, the current study proclaims that other-focused acknowledgments promote the donor's morality preference, and increase subsequent donation desires.

However, according to the morality preference theory, individuals who lack the feeling of doing the morally right thing inhibit subsequent behavioral tendencies. A self-focused acknowledgment emphasizes the positive influence of donors, which can lead to perceptions of personal interest and reduce feelings of doing the morally right thing. Under this condition, donors are prone to make egoistic self-evaluations of their donations, which inhibits subsequent donation desires. For a self-focused acknowledgment, the admiration of personal characteristics from charities can bring social identity, praise, and rewards to donors, such as social reputation and status (Dawson, 1988; Belk, 1995; Deci et al., 1999). Therefore, a self-focused acknowledgment is more likely to stimulate donors' perceptions of their interests and reduce donors' moral preferences, which will inhibit donors' donation desires in the future.

H1: An other-focused acknowledgment elicits a more favorable subsequent donation desire than a self-focused acknowledgment.
H2: Morality preference mediates the relationship between acknowledgment type and subsequent donation desires.

### General rationale

**Figure 1:**
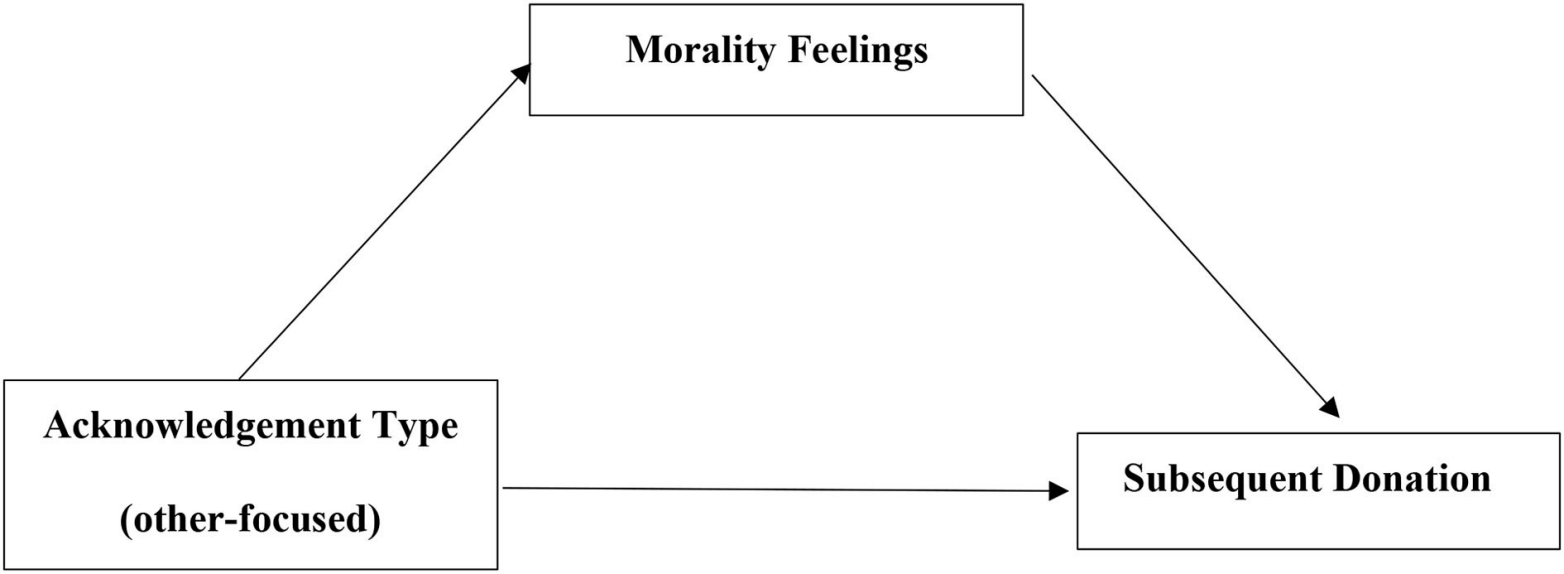
The proposed conceptual model.

The current research proposes that acknowledgment type (self- *v*s. other-focused) (Independent Variables) affects subsequent donation desires (Dependent Variable) through the morality preference (Mediator). Specifically, an other-focused acknowledgment focuses on donation behavior as meaningful and beneficial for the recipient, which increases the donor's morality preference and promotes subsequent donation desires. A self-focused acknowledgment emphasizes the positive personal characteristics of donors, which reduces the donor's morality preference and inhibits subsequent donation desires. For the proposed conceptual model, see Figure 1.

## Methods

### Study 1

Study 1 tests the basic premise that an other-focused acknowledgment elicits a more favorable donation desire than a self-focused acknowledgment, which supports H1. The design of this study is a between-subjects design (other-focused, self-focused, control). The current research also included several tests to exclude the effect of acknowledgment type (positive or negative; intuitive or rational).

#### Participants

Based on the calculation method adopted by Cohen (1977) (effect size *f* = 0.25 and expected power = 0.80), G^*^Power 3.1 software was used to calculate the planned sample size (more than 159 people). Therefore, this study recruited 180 donors from a public university in China to complete a series of experiments. Each participant was randomly assigned to three groups. The final sample size was 168 (female 48.81%, age 20 to 37, *M* = 23.80, *SD* = 3.76), and the size of each group was n_other−focused_= 56, n_self−focused_ = 55, n_control_ = 57.

#### Pretest

The authors employed the two acknowledgment types in study 1: the other-focused condition (“Your donation plays an important role in helping poor children in remote areas. Thank you very much for your donation!”) and the self-focused condition (“We greatly appreciate your kindness and generosity. Thanks very much for your donation!”). To ensure the rationality of the manipulation on acknowledgment type in study 1, the authors selected participants online (*N* = 85, age 25–38, *M* = 27.94, *SD* = 3.19, female 51.76%) for a pretest. Participants were randomly assigned to two groups (other-focused group, self-focused group). The researcher presented the relevant type of acknowledgment for each group. The participants reported their understandings of the target acknowledgment (7-point scales, “the acknowledgment focuses on the benefits to others of donation behaviors,” “the acknowledgment focuses on the positive characteristics of the donor,” 1 = strongly disagree, 7 = strongly agree).

The results showed that the other-focused group reported a higher other-focused dimension than the self-focused group (*M*_other−focused_ = 5.67, *SD* = 0.61; *M*_self−focused_= 2.07, *SD* = 0.64; *t* = 26.65, *df* = 83, *p* < 0.001). The self-focused group reported a higher self-focused dimension than the other-focused group (*M*_self−focused_ = 5.07, *SD* = 0.60; *M*_other−focused_= 1.72, *SD* = 0.67; *t* = 24.33, *df* = 83, *p* < 0.001). The results guaranteed the rationality of the manipulation of acknowledgment in study 1.

#### Procedure

In the main experiment, the researchers raised money from each donor at a university (all donations came from donors) for beneficiaries (children living in remote mountainous areas) who had financial problems. The basic information of the recipients appeared on a computer. Then, the experimenter recorded the information of 180 donors, including their names, addresses, e-mail, and cell phone numbers, and randomly assigned them to three groups (other-focused group, self-focused group, or control group). After 5 days, different acknowledgment letters were sent to the donors based on their group. The other-focused group received the letter saying “Your donation plays an important role in helping poor children in remote areas. Thank you very much for your donation!”. The self-focused group obtained letters saying “We greatly appreciate your kindness and generosity. Thanks very much for your donation!”. The control group received the letter saying “Thank you very much. Best wishes to you!”.

Subsequently, the experimenter sent e-mail invitations to the donors to participate in an online survey. The online survey asked if the donors had received a thank you letter (yes or no). Then, the survey indicated that the charity would hold another donation drive in 10 days and asked if the participants were willing to donate again using a seven-point scale (seven-point scale, 1 = “no desire,” 7 = “will surely donate”). The key questions appeared interspersed with irrelevant items about the impression of the beneficiaries, donation reasons, personal interest, and activity suggestions, among others. Finally, participants were asked to evaluate the contents of the acknowledgment (the same as the pretest); the type (positive or negative) (two items, *a* = 0.92) of the acknowledgment (seven-point scale, 1 = “does not agree at all,” 7 = “agree completely,” “please report your liking of the acknowledgment,” “please report your anger toward the charitable organization”) (Erlandsson et al., 2018); the type (intuitive or rational) (two items, *a* = 0.96) of the acknowledgment (seven-point scale, 1 = “does not agree at all,” 7 = “agree completely,” “the acknowledgment mainly triggers intuitive feelings, such as empathy,” “the acknowledgment mainly triggers rational reactions, such as the thinking of efficiency and effectiveness of charity”) (Bergh and Reinstein, 2020); and to guess the purpose of this survey. After completing all the experiments, the researcher informed the participants of the actual purpose of study 1 and refunded all donations.

#### Results

##### Manipulation check

Twelve participants did not respond. No respondents correctly guessed the purpose of the online survey. See Table 1 for more results of the manipulation check. These results suggested a successful manipulation in study 1.

**Table 1:** Study 1: manipulate check.

	**Other-focused**	**Self-focused**	**Control**	**F**
	**M + SD**	**M + SD**	**M + SD**	
Other focused dimension	5.41, 0.78	2.15, 0.83	3.98, 0.61	*F* = 268.34
Self focused dimension	1.64, 0.59	5.18, 0.67	4.00, 0.65	*F* = 444.55
The liking	4.20, 0.64	4.04, 0.77	3.91, 0.74	*F* = 2.22
The anger	2.54, 0.74	2.42, 0.74	2.37, 0.82	*F* = 0.71
Intuitive dimension	4.30, 0.81	4.18, 0.96	4.21, 0.96	*F* = 0.27
Rational dimension	3.27, 0.77	3.07, 0.72	3.12, 0.89	*F* = 0.90

##### Subsequent donation desires

Significant differences in the subsequent donation desires arose among the three groups (*F* = 75.51, *p* < 0.001). Participants who received an other-focused letter expressed greater subsequent donation desires than those in the self-focused group (*M*_other−focused_ = 5.48, *SD* = 0.95; *M*_self−focused_= 3.35, *SD* = 0.89; *t* = 12.24, *df* = 165, *p* < 0.001) and control group (*M*_control_= 4.26, *SD* = 0.92; *t* = 7.05, *df* = 165, *p* < 0.001). The control group also reported greater donation desires than the self-focused group (*t* = 5.28, *df* = 165, *p* < 0.001).

Multigroup comparisons of the means were carried out by the one-way analysis of variance (ANOVA) test with *post-hoc* contrasts by the Student–Newman–Keuls test. The statistical significance for all tests was set at *p* < 0.05.

##### These findings support H1

Acknowledgment type (self-other focused) affects donors' subsequent donation desires. An other-focused acknowledgment letter elicits greater subsequent donation desires than a self-focused acknowledgment letter. Therefore, it would appear that charities should offer other-focused acknowledgment letters to promote subsequent donation desires.

To improve the external validity of study 1, real gratitude letters from charity organizations were used as a stimulus. Therefore, there existed differences in the language structure among the gratitude letters of the three groups. To eliminate the influence of language structure (Capraro et al., 2022), the current research adopted consistent linguistic expressions on the gratitude letters of the three groups in study 4.

### Study 2

Study 2 verified the mediating role of morality preference, which supported H2. Because prior research only focused on moral behavior preference and did not conduct a quantitative scale on morality preference, study 2 used the feelings of morality as a measurement index to test the mediating role of morality preferences. Study 2 also included several tests to exclude the effect of acknowledgment type (positive or negative; intuitive or rational).

#### Participants

Based on the calculation method adopted by Cohen (1977) (the effect size *d* = 0.5 and the expected power = 0.80), G^*^Power 3.1 software was used to calculate the planned sample size (more than 128 people). This study recruited 150 donors from a public university in China to complete a series of experiments. Each participant was randomly assigned to two groups. The final sample size was 141 (female 48.94%, age 20 to 33, *M* = 23.32, *SD* = 3.07), and the size of each group was *n*_other−focused_= 69, *n*_self−focused_ = 72.

#### Procedure

The authors cooperated with the Young Volunteers Association to raise donations for the beneficiaries (the same as study 1). Donors who were willing to participate in the following online survey were selected. They were randomly arranged into 2 groups (other-focused, self-focused). After donating, participants received the relevant acknowledgment 5 days later (the same as study 1).

Subsequently, the experimenter sent e-mail invitations to the donors to participate in an online survey. Other-focused letters highlighting the beneficial results for the recipients are more likely to convey that “donations reach beneficiaries” than self-focused letters. To rule out this effect, the researcher informed the donors that the beneficiaries had received the donations, along with a photo of the beneficiaries receiving the donations in the email. Then, the online survey asked if the donors had received a thank you letter (yes or no) and reported the feeling of doing morally right things (seven-point scale, 1 = “does not agree at all,” 7 = “agree completely,” “this donation behavior triggers feelings of doing morally right things,” one item). Then, the survey indicated that the charity would hold another donation drive in 10 days and asked if the participants were willing to donate again using a 7-point scale (7-point scale, 1 = “no desire,” 7 = “will surely donate”). To exclude the influence of other mediating effects, the participants also reported emotional reactions (six items, *a* = 0.92), perceived impact (three items, *a* = 0.94), and perceived responsibility (three items, *a* = 0.92) (7-point scale, 1 = “does not agree at all,” 7 = “agree completely”) (Erlandsson et al., 2015).

The key questions appeared interspersed with irrelevant items, such as the impression of the beneficiaries, donation reasons, personal interest, and activity suggestions, among others. Finally, the donors were asked to evaluate the contents of the acknowledgment (other-self focused) and the type (positive or negative/intuitive or rational) of the acknowledgment. They also reported the degree to which the donors believed that the beneficiaries had received the donations (7-point scale, 1 = “0%,” 7 = “100%”) and guessed the purpose of this online survey. After completing all the experiments, the researcher informed the participants of the actual purpose of study 2 and refunded all donations.

#### Results

##### Manipulation check

Nine participants did not respond, and no participants correctly guessed the purpose of the survey. For more results of the manipulation check, see Table 2. These results suggested a successful manipulation in study 2.

**Table 2:** Study 2: Manipulation check.

	**Other-focused**	**Self-focused**	**T**	**P**	**DF**
	**M + SD**	**M + SD**			
Perceived focus on others	5.43 (0.88)	2.28 (0.84)	*t* = 21.73	*p* < 0.001	139
Perceived focus on self	1.91 (0.68)	5.29 (0.68)	*t* = 29.47	*p* < 0.001	139
Liking of appeal	5.13 (0.66)	5.10 (0.73)	*t* = 0.28	*p* = 0.779	139
Anger toward organization	2.16 (0.68)	1.99 (0.72)	*t* = 1.47	*p* = 0.144	139
Perceived appeal to intuition	4.33 (0.61)	4.15 (0.80)	*t* = 1.50	*p* = 0.135	139
Perceived appeal to rationality	3.25 (0.79)	3.04 (0.74)	*t* = 1.59	*p* = 0.115	139
Believed to have received donations	5.07 (0.77)	5.24 (0.80)	*t* = 1.24	*p* = 0.218	139

##### Morality preference (feelings of doing the morally right thing)

The two groups exhibited a significant difference in feelings about doing morally right things. The other-focused group reported a greater feeling of doing morally right things than the self-focused group (*M*_other−focused_ = 4.90, *SD* = 0.94; *M*_self−focused_= 3.42, *SD* = 0.92; *t* = 9.48, *df* = 139, *p* < 0.001).

##### Subsequent donation desires

Significant differences in the subsequent donation desires also arose between the other-focused group and the self-focused group. The other-focused group expressed a greater desire to provide a subsequent donation (*M*_other−focused_ = 5.33, *SD* = 0.97) than the self-focused group (*M*_self−focused_ = 3.82, *SD* = 1.03, *t* = 9.02, *df* = 139, *p* < 0.001). These findings supported H1.

##### Mediation analysis

**Figure 2:**
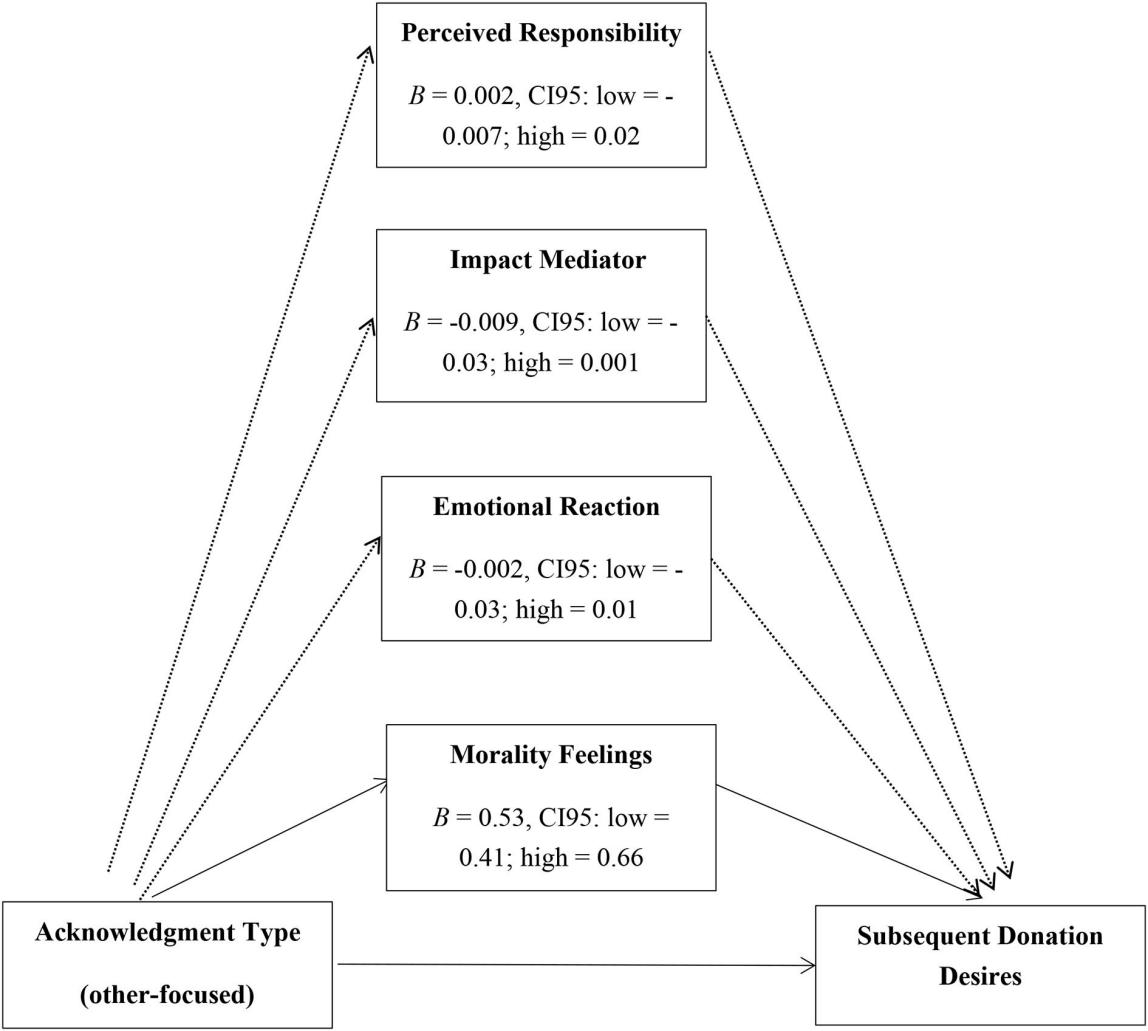
Mediation analysis in study 2.

To test mediation with multiple mediators, we used an SPSS Macro suggested by Preacher and Hayes (2008). The manipulation on acknowledgment type (self-other focused) significantly influenced feelings of morality (feelings of doing the morally right thing) (*B* = 0.63, *SE. B* = 0.07, *t* = 9.48, *p* < 0.001) but not emotional reactions (*B* = 0.03, *SE. B* = 0.11, *t* = 0.27, *p* = 0.789), perceived impact (*B* = 0.16, *SE. B* = 0.11, *t* = 1.51, *p* = 0.133), or perceived responsibility (*B* = 0.05, *SE. B* = 0.11, *t* = 0.43, *p* = 0 0.670). In addition, morality feelings predicted subsequent donation desires even after controlling for other mediators (*B* = 0.53, *SE. B* = 0.06, *p* < 0.001). Confidence intervals from the bootstrap analysis did not include zero for the morality feelings mediator (CI95: low = 0.41; high = 0.66), but did so for the emotional reaction mediator (*B* = −0.003, *SE. B* = 0.01, *p* > 0.05, CI95: low = −0.03; high = 0.01), the perceived impact mediator (*B* = −0.009, *SE. B* = 0.009, *p* > 0.05, CI95: low = −0.03; high = 0.001), and for the perceived responsibility mediator (*B* = 0.002, *SE. B* = 0.007, *p* > 0.05, CI95: low = −0.007; high = 0.02). This indicated that feelings of morality, but not emotional reactions, perceived impact, or perceived responsibility, uniquely mediated the influence of acknowledgment type on subsequent donation desires (see Figure 2).

Study 2 investigated the mediating role of feelings of morality in the relationship between acknowledgment type and subsequent donation desires, constructing an integrative model of the main effect and supporting H2. To verify this mediation effect, study 3 developed a comprehensive scale on morality preference and employed the framing effect to manipulate individuals' morality preferences.

### Study 3

To verify the mediating role of morality preference in a further step, study 3 manipulated the moral value on “what is the morally right thing of donation” and developed a new scale to measure morality preference.

#### Participants

Based on the calculation method adopted by Cohen (1977) (the effect size *f* = 0.25 and the expected power = 0.80), G^*^Power 3.1 software was used to calculate the planned sample size (more than 179 people). This study recruited 210 donors from a public university in China to complete a series of experiments. Each participant was randomly assigned to a 2 (self-focus acknowledgment, other-focus acknowledgment) ^*^ 2 (self-focus frame, other-focus frame) experimental design. The final sample size was 188 (female 47.87%, age 18 to 36, *M* = 23.63, *SD* = 3.66), and the size of each group was *n*_selfself_ = 51, *n*_selfother_ = 44, *n*_otherself_ = 48, *n*_otherother_ = 45.

#### Pretest

This study used two frames on “what is the morally right thing of donation.” One emphasized that “Donation makes yourself better. Focusing on yourself is the morally right thing in donation.” The other stressed that “Donation makes others better. Focusing on others is the morally right thing in donation.” To testify the framing effect, the authors selected participants online (*N* = 82, age 18 to 38, *M* = 23.16, *SD* = 3.93, female 51.22%) for a pretest. Participants were randomly assigned to two groups (other-group, self-group). The participants in each group received relevant information and reported their moral value of the morally right thing in donation (7-point scales, 1 focusing on self is the morally right thing in donation, 7 focusing on others is the morally right thing in donation). The results showed that the other-group reported a higher result than the self-group (*M*_other−focused_ = 4.65, *SD* = 0.98; *M*_self−focused_ = 3.64, *SD* = 1.08; *t* = 4.43, *df* = 80, *p* < 0.001), which verified the viability of the manipulation in study 3.

The authors employed the two acknowledgment types with consistent linguistic expressions in study 3: the other-focused condition (“Thanks very much for your donation. Your help brings a better future to others.”); and the self-focused condition (“Thanks very much for your donation. Your help brings a better future to yourself.”). The authors selected participants online (*N* = 78, age 18 to 34, *M* = 23.05, *SD* = 2.88, female 44.87%) for a pretest. Participants were randomly assigned to two groups (other-focused group, self-focused group) and obtained the relevant type of acknowledgment. The participants in each group reported their understanding of the target acknowledgment (7-point scales, “the acknowledgment focuses on the influence of donation behaviors to others,” “the acknowledgment focuses on the influence of donation behaviors to self,” 1 = strongly disagree, 7 = strongly agree).

The results showed that the other-focused group reported a higher other-focused dimension than the self-focused group (*M*_other−focused_ = 5.15, *SD* = 0.95; *M*_self−focused_ = 2.58, *SD* = 1.11; *t* = 11.04, *df* = 76, *p* < 0.001). The self-focused group also reported a higher self-focused dimension than the other-focused group (*M*_self−focused_ = 5.00, *SD* = 0.93; *M*_other−focused_ = 1.95, *SD* = 0.93; *t* = 14.46, *df* = 76, *p* < 0.001). The results verified the viability of the manipulation of acknowledgment in study 3.

Because prior research did not conduct a scale on morality preference, study 3 developed a new scale on morality preference according to the morality preference theory. The scale consisted of three items: “To what extent do you think this is the morally right thing to do,” “To what extent do you feel doing the morally right thing,” “To what extent do you prefer moral behavior” (7-point scale, 1 0%, 7 100%). To ensure the reliability and validity of this scale, the authors selected 200 individuals who had volunteered during the COVID-19 pandemic for a pretest (female 54.50%, age 18–30, *M* = 21.76, *SD* = 2.45). The data showed that the reliability of the morality preference scale was 0.87, and the half split reliability was 0.82. The commonality of each factor was no <0.74, and the load was no <0.88.

#### Procedure

This study raised money through a university in China that cooperated with the Volunteers Association. The researcher recruited 210 donors from a university in China who had donated money to the beneficiaries (the same as study 1). Afterward, the researcher invited these donors to participate in a subsequent online survey and arranged them into a 2 (self-focus acknowledgment, other-focus acknowledgment) ^*^ 2 (self-focus frame, other-focus frame) experimental design. After 7 days, participants received the relevant frame information and acknowledgment letters (see pretest).

Subsequently, the donors received invitations to participate in an online survey. The researcher informed the donors that all the beneficiaries had received the donations, along with a photo of the beneficiaries receiving the donations in the email. Similar to previous studies, this survey asked donors if they had received the frame information and acknowledgment letter, and reported their morality preference after reading the acknowledgment letter and frame information. The donors also indicated the possibility of participating in a subsequent donation activity 10 days later, using the same scale as in previous studies. Finally, the donors were asked to evaluate the contents of the acknowledgment and frame information (other-focused or self-focused), the type (positive or negative) of the acknowledgment, and the type (intuitive or rational) of the acknowledgment. They also reported the extent to which they believed that the beneficiaries had received donations and guessed the purpose of this online survey. After completing all the experiments, the researcher informed the participants of the actual purpose of study 3 and refunded all donations.

#### Results

##### Manipulation check

Twenty-two donors did not respond, and no participants correctly guessed the purpose of the survey. For more results of the manipulation check, see Table 3. These results suggested a successful manipulation in study 3.

**Table 3:** Study 3: Manipulation check.

	**Self-self**	**Self-other**	**Other-self**	**Other-other**	**F**	***P***
	**M + SD**	**M + SD**	**M + SD**	**M + SD**		
Perceived focus on others (acknowledgment)	1.90 (0.94)	1.95 (0.81)	5.35 (0.81)	5.02 (0.89)	223.52	<0.001
Perceived focus on self (acknowledgment)	5.47 (0.86)	5.32 (0.91)	2.00 (0.83)	2.27 (0.96)	213.54	<0.001
Perceived focus on others (frame)	2.06 (0.83)	5.25 (0.78)	2.23 (0.78)	5.11 (0.88)	214.58	<0.001
Perceived focus on self (frame)	5.04 (0.92)	2.34 (0.83)	5.04 (0.90)	2.33 (0.85)	148.22	<0.001
Liking of appeal	4.73 (0.67)	4.82 (0.79)	4.85 (0.77)	4.73 (1.16)	0.26	0.853
Anger toward organization	1.88 (0.59)	1.93 (0.66)	1.94 (0.70)	1.98 (0.62)	0.18	0.911
Perceived appeal to intuition	4.45 (0.70)	4.61 (0.84)	4.40 (0.71)	4.42 (0.84)8	0.72	0.539
Perceived appeal to rationality	3.78 (0.86)	3.59 (0.73)	3.77 (0.69)	3.53 (0.84)	1.23	0.300
Believed to have received donations	5.25 (0.59)	5.16 (0.83)	5.33 (0.81)	5.20 (0.89)	0.43	0.734

##### Morality preference

The findings revealed a significant interaction of acknowledgment type and frame information on morality preference (*F* = 72.84, *p* < 0.001). When the frame was self-focus, participants receiving self-focused acknowledgment reported higher morality preference than participants obtaining other-focused acknowledgment (*M*_other−focused_ = 3.58, *SD* = 0.74; *M*_self−focused_ = 4.49, *SD* = 0.78; *t* = 5.91, *df* = 97, *p* < 0.001). However, when the frame was other-focus, participants gaining other-focused acknowledgment reported higher morality preference than participants getting self-focused acknowledgment (*M*_other−focused_ = 4.44, *SD* = 0.72; *M*_self−focused_ = 3.45, *SD* = 0.79; *t* = 6.16, *df* = 87, *p* < 0.001).

##### Subsequent donation desire

The results revealed a predicted interactive effect of acknowledgment type and frame information on subsequent donation desires (*F* = 87.45, *p* < 0.001). When the frame was self-focus, participants receiving self-focused acknowledgment reported higher donation desires than participants obtaining other-focused acknowledgment (*M*_other−focused_ = 3.90, *SD* = 0.86; *M*_self−focused_ = 5.02, *SD* = 0.86; *t* = 6.51, *df* = 97, *p* < 0.001). However, when the frame was other-focus, participants gaining other-focused acknowledgment reported higher donation desires than participants getting self-focused acknowledgment (*M*_other−focused_ = 5.16, *SD* = 0.82; *M*_self−focused_ = 3.77, *SD* = 0.83; *t* = 11.04, *df* = 76, *p* < 0.001).

##### Moderated mediation analysis

Because the moderator (frame information) in study 3 was hypothesized to influence the dependent variable (subsequent donation desires) through the mediator (morality preference), the data were submitted to a moderated mediation analysis (using the macro PROCESS, model 8, with 5,000 bootstrapping resamples; see Hayes, 2013).

The results revealed that the moderating effect of frame information [*B* = 1.55; 95% CI = (lower bound 1.16, upper bound 1.98)] was significant (95% CI did not contain 0). Specifically, the results verified a significant interactive effect (95% CI did not contain 0) of acknowledgment type and frame information on morality preference [*B* = 1.90; 95% CI = (lower bound 1.46, upper bound 2.34); *t* = 8.53, *p* < 0.001]. The influence of morality preference on subsequent donation desires was significant (95% CI did not contain 0) [*B* = 0.82; 95% CI = (lower bound 0.71, upper bound 0.93); *t* = 14.68, *p* < 0.05]. These findings indicated that the mediation effect of morality preference was moderated by frame information. When the frame was other-focus, an other-focused acknowledgment letter triggered greater morality preference and subsequent donation desires than a self-focused acknowledgment letter [*B* = −0.81; 95% CI = (lower bound −1.09, upper bound −0.54)] (95% CI did not contain 0). When the frame was self-focus, a self-focused acknowledgment letter elicited greater morality preference and subsequent donation desires than an other-focused acknowledgment letter [*B* = 0.74; 95% CI = (lower bound 0.49, upper bound 1.04)] (95% CI did not contain 0).

Study 3 manipulated the morality preference through frame information, which moderated the relationship between acknowledgment type and subsequent donation desires. The study used consistent linguistic expressions of acknowledgment and developed a relevant scale on morality preference, which verified the mediating role of morality preference in a further step. Study 4 employed consistent linguistic expressions of gratitude letters in a real donation context.

### Study 4

Study 4 adopted consistent linguistic expressions on the gratitude letters of the three groups (other-focused, self-focused, control) to eliminate the influence of language structure (Capraro et al., 2022) in a real donation context. Study 4 also used subsequent donation amount (the average, ¥) as the dependent variable to improve the internal validity.

#### Participants

Based on the calculation method adopted by Cohen (1977) (effect size *f* = 0.25 and expected power = 0.80), G^*^Power 3.1 software was used to calculate the planned sample size (more than 159 people). Therefore, this study recruited 180 donors from a public university in China to complete a series of experiments. Each participant was randomly assigned to three groups. The final sample size was 163 (female 50.92%, age 18 to 32, *M* = 22.37, *SD* = 2.81), and the size of each group was *n*_other−focused_= 54, *n*_self−focused_ = 53, *n*_control_ = 56.

#### Procedure

In the main experiment, the researchers raised money from each donor at a university for beneficiaries (children living in remote mountainous areas) who had financial problems (the same as study 1). 180 donors were randomly assigned to three groups (other-focused group, self-focused group, or control group). After 7 days, relevant acknowledgment letters were sent to the donors (see study 3). The other-focused group received the letter saying “Thanks very much for your donation. Your help brings a better future to others.” The self-focused group obtained letters saying “Thanks very much for your donation. Your help brings a better future to yourself.” The control group received the letter saying “Thanks very much for your donation.”

Subsequently, the experimenter sent e-mail invitations to the donors to participate in an online survey. The online survey asked if the donors had received a thank you letter (yes or no), and reported the feeling of doing morally right things. The survey also included a link to an online donation drive (for children living in remote mountainous areas) and invited the participants to donate again. The key questions appeared interspersed with irrelevant items about the impression of the beneficiaries, donation reasons, personal interest, and activity suggestions, among others.

Finally, participants were asked to evaluate the contents of the acknowledgment; the type (positive or negative) (two items, *a* = 0.92) of the acknowledgment (Erlandsson et al., 2018); the type (intuitive or rational) (two items, *a* = 0.96) of the acknowledgment (Bergh and Reinstein, 2020); and to guess the purpose of this survey. After completing all the experiments, the researcher informed the participants of the actual purpose of study 4 and refunded all donations.

#### Results

##### Manipulation check

Seventeen participants did not respond. No respondents correctly guessed the purpose of the online survey. See Table 4 for more results of the manipulation check. These results suggested a successful manipulation in study 4.

**Table 4:** Study 4: Manipulate check.

	**Other-focused**	**Self-focused**	**Control**	**F**
	**M + SD**	**M + SD**	**M + SD**	
Other focused dimension	5.67, 1.08	2.11, 0.91	4.39, 0.87	*F* = 188.95
Self focused dimension	1.80, 0.86	5.34, 1.06	4.21, 0.71	*F* = 226.94
The liking	4.17, 0.72	4.23, 0.82	4.21, 0.89	*F* = 0.08
The anger	2.59, 1.04	2.62, 1.06	2.48, 1.04	*F* = 0.27
Intuitive dimension	4.20, 0.83	4.32, 0.87	4.52, 0.99	*F* = 1.71
Rational dimension	3.50, 0.95	3.38, 0.97	3.48, 1.08	*F* = 0.24

##### Morality feelings (feelings of doing the morally right thing)

The three groups exhibited a significant difference in feelings about doing morally right things (*F* = 49.09, *p* < 0.001). The other-focused group reported a greater feeling of doing morally right things than the self-focused group (*M*_other−focused_ = 5.07, *SD* = 0.77; *M*_self−focused_= 3.45, *SD* = 0.87; *t* = 9.84, *df* = 160, *p* < 0.001), and control group (*M*_control_= 4.11, *SD* = 0.91; *t* = 5.95, *df* = 160, *p* < 0.001). The control group also reported a higher feeling of doing morally right things than the self-focused group (*t* = 4.01, *df* = 160, *p* < 0.001). Multigroup comparisons of the means were carried out by the ANOVA test with *post-hoc* contrasts by the Student–Newman–Keuls test. The statistical significance for all tests was set at *p* < 0.05.

##### Subsequent donation amount

Significant distinctions in the subsequent donation amount appeared among the three groups (*F* = 15.59, *p* < 0.001). The other-focused group donated more than those in the self-focused group (*M*_other−focused_ = 11.20, *SD* = 7.77; *M*_self−focused_= 3.96, *SD* = 5.58; *t* = 5.58, *df* = 160, *p* < 0.001) and control group (*M*_control_= 7.32, *SD* = 6.60; *t* = 3.03, *df* = 160, *p* < 0.001). The control group also reported greater donation amount than the self-focused group (*t* = 2.61, *df* = 160, *p* < 0.001). Multigroup comparisons of the means were carried out by the ANOVA test with *post-hoc* contrasts by the Student–Newman–Keuls test. The statistical significance for all tests was set at *p* < 0.05.

Study 4 further tested the mediating role of moral feelings in the relationship between acknowledgment type and subsequent donation amount. As predicted, the results of a bootstrapping analysis using PROCESS model 4 (with 5,000 bootstrapping resamples; see Hayes, 2013) found a significant indirect effect of acknowledgment type on subsequent donation amount through the morality feelings (95% CI did not contain 0) [*B* = 5.21; 95% CI = (lower bound 4.23, upper bound 6.53)]. The results revealed that the effect of acknowledgment type on subsequent donation amount was mediated by the morality feelings.

The findings showed that acknowledgment type (self-other focused) affected donors' subsequent donations. An other-focused acknowledgment letter elicits a greater subsequent donation amount than a self-focused acknowledgment letter. This main effect was not influenced by language structure.

## General discussion

### Conclusion

The current research explores the influence of acknowledgment type (other-self focused) on donors' subsequent donation desires. Study 1 finds that the acknowledgment type can affect donors' subsequent donation desires. An other-focused acknowledgment letter elicits more positive desires to make subsequent donations than a self-focused acknowledgment letter. Study 2 reveals the mediating role of the feelings of doing the morally right thing in the relationship between the acknowledgment type and subsequent donation desires, which constructs an integrative model of the main effect. Study 3 manipulates the moral value on “what is the morally right thing of donation” to verify the mediating effect of morality preference in a further step. Study 4 eliminates the influence of language structure among acknowledgment letters and verifies the main effect in a real donation behavior context.

### Theoretical contributions

First, prior studies ignore the influence of the acknowledgment type (other-self focused) on subsequent donation desires. The current research enriches relevant studies in the research field of acknowledgment. It provides theoretical foundations and practical suggestions for the management and administration of charities. This research shows that the positive effects of expressing gratitude are moderated by acknowledgment type, which potentially enriches the existing relevant literature. However, if charities want to sustain donor loyalty, the type of acknowledgment is crucially important. The findings first distinguish other-focused acknowledgments and self-focused acknowledgments, construct an integrative model for the proposed hypothesis, and provide effective suggestions for charities.

Prior studies reveal that the effects of acknowledgments on subsequent donation desires are still uncertain (Prince and File, 1994). Some donors deliberately employ anonymity to avoid acknowledgments from charities, while others are depressed after receiving acknowledgments (Prince and File, 1994). Based on morality preference theory, the current study offers an explicit explanation of donors' complex attitudes toward acknowledgments, which remedies the existing theoretical gap and expands the research field. Based on the morality preference theory, an other-focused acknowledgment emphasizes the altruistic benefits of donation behaviors and provides altruistic perceptions for the donor, which increases the donor's feeling of doing the morally right thing and subsequent prosocial behavior. However, a self-focused acknowledgment emphasizes the positive influences on donors, which can lead to perceptions of personal interest and reduce feelings of doing the morally right thing. Under this condition, donors are prone to decrease subsequent donations. From the perspective of morality preference, the current study provides precise predictions on the influence of acknowledgment type on the desire to make donations, which develops a new research direction.

Third, by demonstrating the influence of feelings regarding moral behavior on subsequent donation desires, the current study reveals the distinction between an other-focused acknowledgment letter and a self-focused letter and presents a novel approach for charities to increase the desire by donors to make additional donations. This research also expands the literature on morality preference theory by specifying behavioral consequences and analyzing the psychological process within a philanthropic context.

This study also explores the influences of other- and self-focused frames on donation desires in the acknowledgment context. Although prior research in the area of charitable appeals tests the influences of other- vs. self-focused appeals, these studies do not distinguish previous non-donors from previous donors (Bendapudi et al., 1996; Brunel and Nelson, 2000; Ferguson et al., 2008). People may provide donations because of external stimuli (self-interests) or internal motivation (altruistic motives). Other- *vs*. self-focused appeals may trigger different motivations that lead to varying donation behavior (Brunel and Nelson, 2000; Fisher et al., 2008; White and Peloza, 2009; Feiler et al., 2012). However, an analysis of acknowledgments only focuses on previous donors and subsequent donation desires, which provides a different context for other- vs. self-focused frames. The study also found that only an other-focused acknowledgment triggers internal motivation (the feeling of doing the morally right thing) and improves subsequent donation performance. A self-focused acknowledgment elicits external motivation (self-interests) and inhibits subsequent donation performance.

### Limitation and future research

The study investigates the influence of acknowledgment type on donation desires from the perspective of morality preference. Previous studies find that social rewards as external motivation can also influence donation behavior under some particular conditions (Fisher and Ackerman, 1998; Winterich et al., 2013a). The current study mainly focuses on the perspective of intrinsic motivation and does not compare internal motivation and external motivation. Further research can explore the distinction between intrinsic and external motivations for donation behavior. Furthermore, the dimension of moral identity may moderate the relationship between acknowledgment type and subsequent donation. Aquino and Reed (2002) divide moral identity into two dimensions. Internalization reflects the extent to which moral characteristics are central to an individual's self-concept, whereas symbolization reflects the extent to which individuals publicly express moral behavior and communicate moral values through non-verbal behaviors in their daily lives. The internalization dimension directly draws on the self-importance of moral characteristics. An other-focused acknowledgment is better to meet the needs to keep consistency with moral values than a self-focused acknowledgment. The symbolization dimension draws on a more general sensitivity to the moral self as a social object. A self-focused acknowledgment leads to more attention on social identity which is more suitable than an other-focused acknowledgment. Other possible moderators may also exist, including the characteristics of charities, such as size, reputation, social influence, and the type of publicity (public or private). Future research can also explore boundary conditions for the main effect.

## Data availability statement

The original contributions presented in the study are included in the article/Supplementary material, further inquiries can be directed to the corresponding author.

## Ethics statement

The studies involving human participants were reviewed and approved by Ethics Committee of Center for Psychological Science and Health, China University of Geosciences (Wuhan). The patients/participants provided their written informed consent to participate in this study.

## Author contributions

FW is responsible for outlining and writing article. SX is responsible for the experiments. YZ is responsible for the data analysis. All authors contributed to the article and approved the submitted version.

## Conflict of interest

The authors declare that the research was conducted in the absence of any commercial or financial relationships that could be construed as a potential conflict of interest.

## Publisher's note

All claims expressed in this article are solely those of the authors and do not necessarily represent those of their affiliated organizations, or those of the publisher, the editors and the reviewers. Any product that may be evaluated in this article, or claim that may be made by its manufacturer, is not guaranteed or endorsed by the publisher.
