# Supplementary material for: Self-focused or other-focused: The influence of acknowledgment type on subsequent donation desires
Source: Front Psychol. 2022 Sep 23;13:959369. doi: 10.3389/fpsyg.2022.959369 (PMC9583881; doi:10.3389/fpsyg.2022.959369)
Supplement: Supplementary file 1 [file Data_Sheet_1.docx]

**Appendix**

**Study 1**

**Understandings of the target acknowledgment**

1. point scales, "the acknowledgment focuses on the benefits to others of donation behaviors", "the acknowledgment focuses on the positive characteristics of the donor", 1 = strongly disagree, 7 = strongly agree)

**Subsequent donation desires**

the charity will hold another donation drive in 10 days and report the possibility that you are willing to donate again (seven-point scale, 1 = "no desire", 7 = "will surely donate").

**The type (positive or negative) of the acknowledgment**

(seven-point scale, 1 = “does not agree at all”, 7 = “agree completely”, “please report your liking of the acknowledgment”, “please report your anger toward the charitable organization”)

**The type (intuitive or rational) of the acknowledgment**

(seven-point scale, 1 = “does not agree at all”, 7 = “agree completely”, “the acknowledgment mainly triggers intuitive feelings, such as empathy”, “the acknowledgment mainly triggers rational reactions, such as the thinking of efficiency and effectiveness of charity”)

**Study 2**

**The feeling of doing morally right things**

(seven-point scale, 1 = “does not agree at all”, 7 = “agree completely”, “this donation behavior triggers feelings of doing morally right things”

**Subsequent donation desires**

the charity will hold another donation drive in 10 days and report the possibility that you are willing to donate again (seven-point scale, 1 = "no desire", 7 = "will surely donate").

**Emotional reactions(distress and sympathy), perceived impact, and perceived responsibility**

(7 point scale, 1 = “does not agree at all”, 7 = “agree completely”)

Rate how you feel when reading this version (personal distress) .

1. I feel downhearted

2. I feel sad

3. I feel emotionally uneasy

Rate how you react emotionally when reading this version (sympathy) .

4. I feel intense compassion

5. I feel strong empathic feelings

6. I feel emotionally touched

Rate how you perceive that the utility of donating money is in this version (perceived impact) .

7. I think one can do a lot of good

8. I think it seems possible to make a big difference

9. I believe the expected consequences are very positive

Rate how you consider your personal responsibility in this version (perceived responsibility) .

10. I have a moral obligation to help to the best of my ability

11. I have a personal responsibility to help as much as I can

12. I have a duty to try to help.

**The type (positive or negative) of the acknowledgment**

(seven-point scale, 1 = “does not agree at all”, 7 = “agree completely”, “please report your liking of the acknowledgment”, “please report your anger toward the charitable organization”)

**The type (intuitive or rational) of the acknowledgment**

(seven-point scale, 1 = “does not agree at all”, 7 = “agree completely”, “the acknowledgment mainly triggers intuitive feelings, such as empathy”, “the acknowledgment mainly triggers rational reactions, such as the thinking of efficiency and effectiveness of charity”)

**Understandings of the target acknowledgment**

1. point scales, "the acknowledgment focuses on the benefits to others of donation behaviors", "the acknowledgment focuses on the positive characteristics of the donor", 1 = strongly disagree, 7 = strongly agree)

Please report the degree to which you believe that the beneficiaries have received the donations (7 point scale, 1 = "0%", 7 = "100%")

**Study 3**

**Moral identity scale**

Self-Importance of Moral Identity Scale and Instructions (1 = strongly disagree, 7 = strongly agree; Aquino & Reed, 2002)

Listed alphabetically below are some characteristics that might describe a person: Caring, Compassionate, Fair, Friendly, Generous, Helpful, Hardworking, Honest, Kind

The person with these characteristics could be you or it could be someone else. For a moment, visualize in your mind the kind of person who has these characteristics. Imagine how that person would think, feel, and act. When you have a clear image of what this person would be like, answer the following questions using the scale below (*Note.* I = Internalization item; S = Symbolization item; R = reverse coded.).

1. It would make me feel good to be a person who has these characteristics. (I)

2. Being someone who has these characteristics is an important part of who I am. (I)

3. I often wear clothes that identify me as having these characteristics. (S)

4. I would be ashamed to be a person who had these characteristics. (I/R)

5.The types of things I do in my spare time (e.g., hobbies) clearly identify me as having these characteristics. (S)

6. The kinds of books and magazines that I read identify me as having these

characteristics. (S)"

7. Having these characteristics is not really important to me. (I/R)

8. The fact that I have these characteristics is communicated to others by my membership in certain organizations. (S)

9. I am actively involved in activities that communicate to others that I have these

characteristics. (S)"

10. I strongly desire to have these characteristics. (I)

**The feeling of doing morally right things**

(seven-point scale, 1 = “does not agree at all”, 7 = “agree completely”, “this donation behavior triggers feelings of doing morally right things”

**Subsequent donation desires**

the charity will hold another donation drive in 10 days and report the possibility that you are willing to donate again (seven-point scale, 1 = "no desire", 7 = "will surely donate").

**The type (positive or negative) of the acknowledgment**

(seven-point scale, 1 = “does not agree at all”, 7 = “agree completely”, “please report your liking of the acknowledgment”, “please report your anger toward the charitable organization”)

**The type (intuitive or rational) of the acknowledgment**

(seven-point scale, 1 = “does not agree at all”, 7 = “agree completely”, “the acknowledgment mainly triggers intuitive feelings, such as empathy”, “the acknowledgment mainly triggers rational reactions, such as the thinking of efficiency and effectiveness of charity”)

**Understandings of the target acknowledgment**

1. point scales, "the acknowledgment focuses on the benefits to others of donation behaviors", "the acknowledgment focuses on the positive characteristics of the donor", 1 = strongly disagree, 7 = strongly agree)

Please report the degree to which you believe that the beneficiaries have received the donations (7 point scale, 1 = "0%", 7 = "100%")
